# Proximal Radioulnar Synostosis Post-radial Head Fracture: Surgical Excision and Anconeus Muscle Flap Interposition for Functional Restoration

**DOI:** 10.7759/cureus.82783

**Published:** 2025-04-22

**Authors:** Deepak Singh, Chia Hua Lim, Shalimar Abdullah, Jamari Sapuan, Elaine Zi Fan Soh

**Affiliations:** 1 Hand and Microsurgery Unit, Department of Orthopaedics and Traumatology, Faculty of Medicine, Universiti Kebangsaan Malaysia, Kuala Lumpur, MYS

**Keywords:** anconeus, flap, integra, radioulnar, synostosis

## Abstract

Adult proximal radioulnar synostosis is a rare complication where its manifestation is due to either post-surgery or post-traumatic effect causing limitation in the function of the forearm especially in supination and pronation. Most common occurrences of synostosis over the forearm region can be divided based on anatomical location, which involves the distal third, middle third, and proximal third forearm region. We report the case of a 42-year-old woman who developed proximal radioulnar synostosis following a conservatively treated radial head fracture 18 months earlier. She presented with pain and restricted forearm rotation during heavy lifting. Surgical excision of the synostosis with anconeus muscle flap interposition was performed. Four months postoperatively, the patient demonstrated improved pronation and supination with no further complaints. Surgical intervention should be considered in adult cases where forearm rotation is compromised.

## Introduction

Synostosis refers to the abnormal fusion of two bones, with radioulnar synostosis being an uncommon complication following forearm trauma, reported in 0-9.4% of cases [1]. Vince and Miller's classification divides radioulnar synostosis into three types based on location: distal third, middle third, and proximal third [1]. Hastings and Graham later expanded this classification, identifying six forearm regions prone to synostosis [2]. Additionally, Jupiter and Ring subdivided proximal synostosis into types 3A (distal to the bicipital tuberosity), 3B (at the radial head and proximal radioulnar joint), and 3C (extending to the distal humerus) [3]. Risk factors include postoperative complications and trauma. The incidence of proximal radioulnar synostosis ranges from 1.2% to 6.2% in patients with combined radius and ulna fractures [4]. CT scanning with 3D reconstruction aids in determining the precise location and extent of the synostosis, guiding the surgical approach [5]. Surgical approaches, such as posterolateral or posterior global, are selected based on the extent of bony ankylosis. Pronation and supination range of motion is exclusively seen in the forearm alone. This occurrence is partly due to two condylar articulations (proximal radioulnar joint and distal radioulnar joint) and also an osseoligamentous intercondylar segment. Both axes of the radioulnar joint must be aligned coaxially for an even forearm rotation to take place. Here, we present a case of proximal radioulnar synostosis treated with excision and anconeus muscle flap interposition with Integra application.

## Case presentation

**Figure 1:**
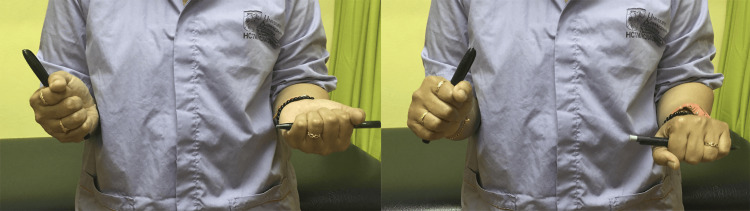
Preoperative clinical image showing limitation in pronation and supination of patient right forearm as compared to the normal contralateral side

A 42-year-old woman presented with a four-month history of pain and limited forearm rotation. Eighteen months earlier, she sustained a radial head fracture from a fall, treated conservatively with casting for one month at another facility. The patient was diagnosed with proximal radioulnar synostosis during follow-up but defaulted on the proposed surgery. Upon presentation to our center post-trauma of 18 months, clinical examination revealed an arc of motion restriction with 20° of pronation and supination was only achievable (Figure 1).

**Figure 2:**
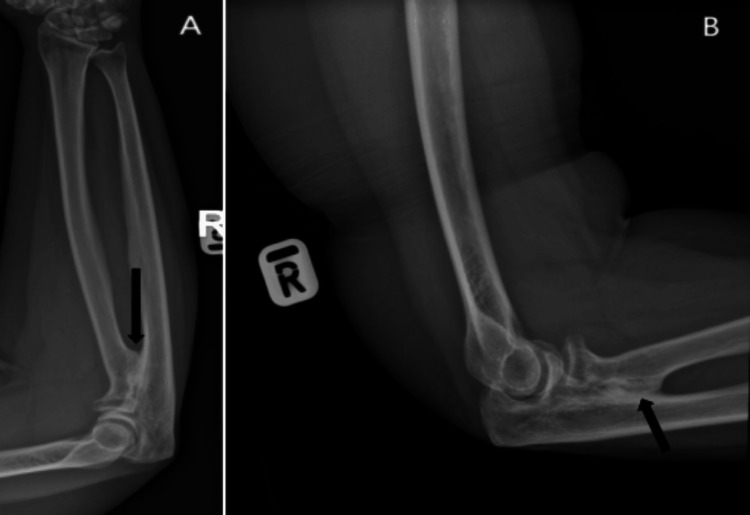
AP view of the right forearm (A) and lateral view of the right elbow (B), with the arrow showing proximal radioulnar synostosis formation AP: anteroposterior

**Figure 3:**
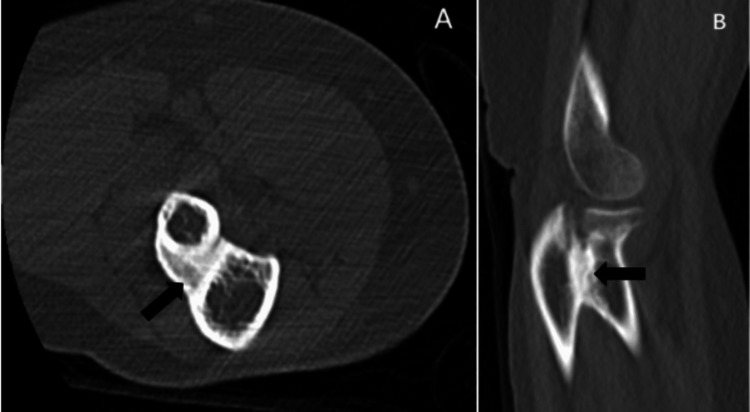
CT scan of the right elbow 18 months after the patient's injury in axial cut (A) and sagittal cut (B), with the arrow indicating the synostosis formation at the PRUJ level measuring about 2 cm and healed radial head fracture PRUJ: proximal radioulnar joint

Radiographs confirmed synostosis at the proximal radioulnar joint with a healed radial head fracture (Figure 2). A CT scan was performed to assess the synostosis extent, measuring 2 cm (Figure 3).

**Figure 4:**
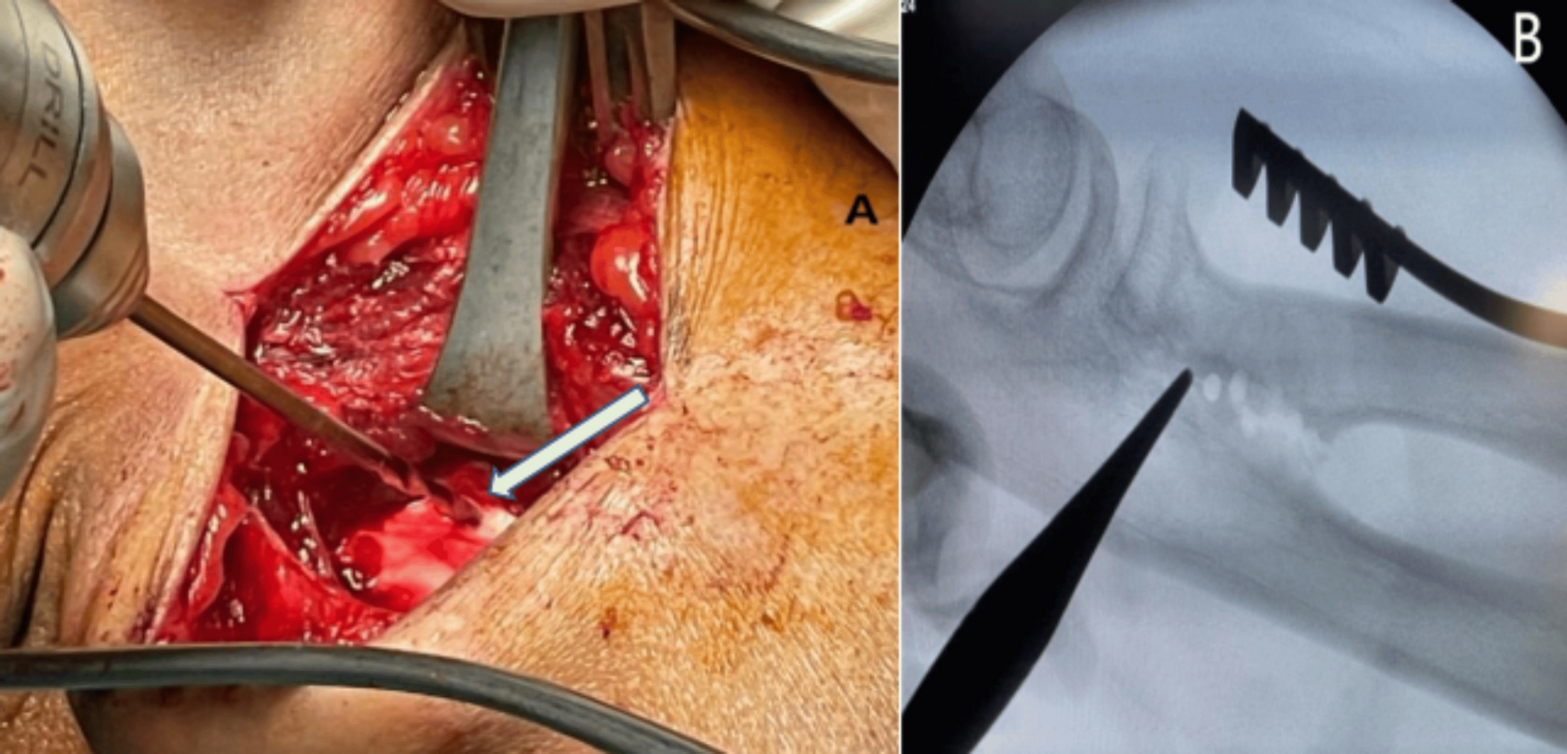
Intraoperative image, with the arrow showing the level of synostosis which was identified to be about 2 cm (A). Intraoperative radiography showing the level of synostosis which was located at the PRUJ level (B) PRUJ: proximal radioulnar joint

**Figure 5:**
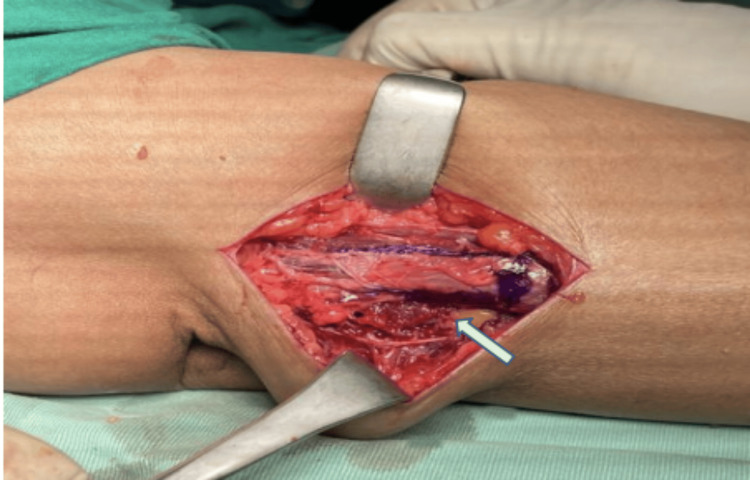
The arrow showing anconeus muscle flap interposition at the level of excised synostosis between the separated radius and ulna

**Figure 6:**
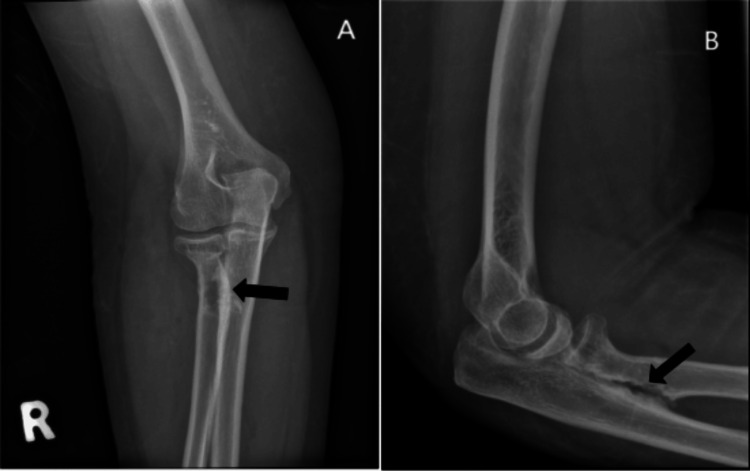
Postoperative radiograph AP view of the right elbow (A) and lateral view of the right elbow (B), with the arrow showing no interval calcification or remnant of ossification AP: anteroposterior

The patient underwent synostosis excision via a posterolateral approach, where a 2 cm synostosis segment was removed (Figure 4). Intraoperatively, post-excision of the synostosis, the forearm was able to fully pronate and supinate. An anconeus muscle flap was interposed between the radius and ulna to prevent recurrence, and Integra was applied (Figure 5). No intraoperative complications were noted, and the patient was discharged after two days without splinting. Postoperative radiographs showed no residual calcification or remnant of ossification noted (Figure 6), and she began physiotherapy one week later.

**Figure 7:**
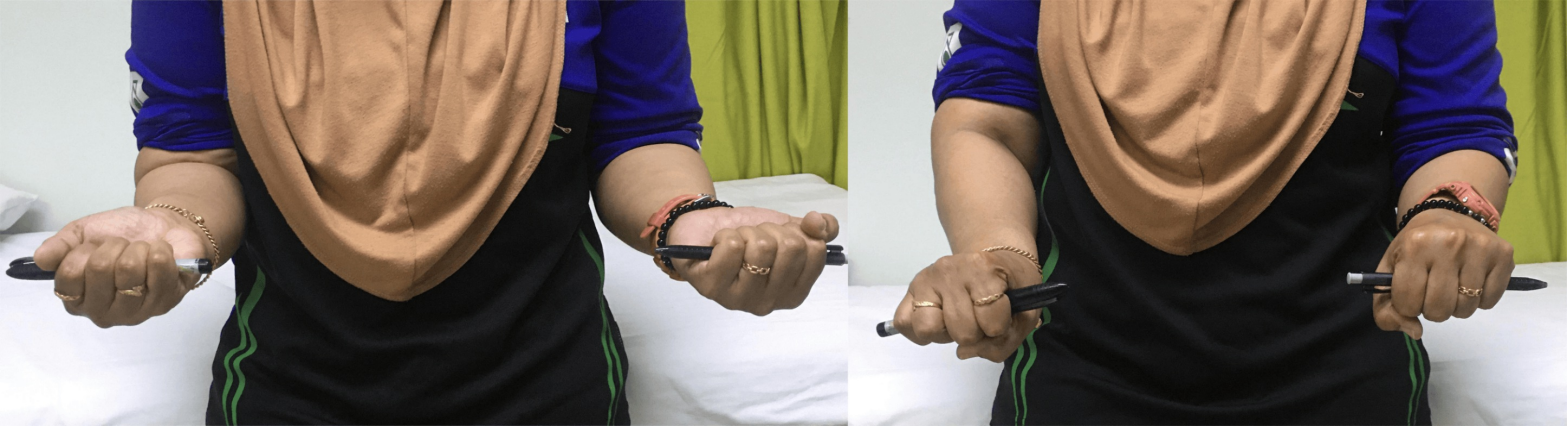
Postoperative fourth-month follow-up, with the clinical image showing improved function in patient right forearm supination and pronation rotation

At her four-month follow-up, the patient reported no pain, with an improved arc of motion: 90° supination and 60° pronation (Figure 7).

**Figure 8:**
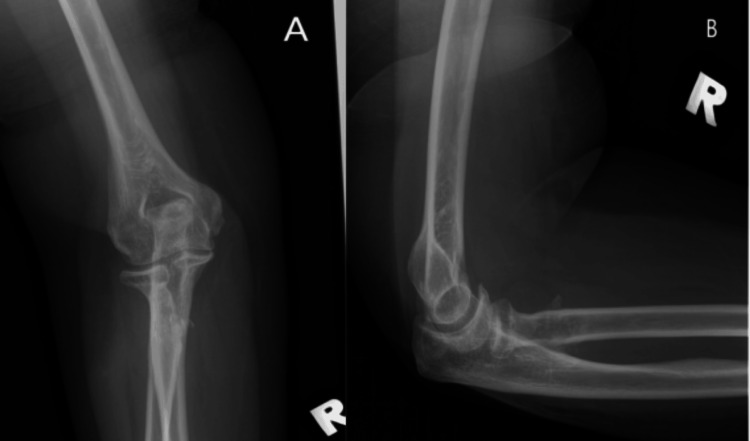
Right elbow radiograph AP view (A) and lateral view (B) four months post-excision showing no new ossification in the PRUJ interval AP: anteroposterior; PRUJ: proximal radioulnar joint

She successfully returned to work, and radiographs confirmed no recurrence of synostosis (Figure 8). The patient, however, defaulted in her further follow-up thereafter. 

## Discussion

Post-traumatic radioulnar synostosis is an uncommon complication, with reported incidence rates ranging from 0% to 9.4% for forearm injuries [1]. Synostosis of the radius and ulna can be either congenital or post-traumatic. Classification systems typically focus on either anatomical location or functional limitation. Vince and Miller categorized forearm synostosis into three types based on location: distal third, middle third, and proximal third [1]. Jupiter and Ring further subdivided the proximal synostosis (type 3) into three subgroups: type 3A (at or distal to the bicipital tuberosity), type 3B (at the radial head and proximal radioulnar joint, as seen in our case), and type 3C (contiguous with heterotopic ossification extending to the distal humerus) [3]. Hastings and Graham introduced a separate classification system addressing functional limitations [2].

Surgical intervention is typically justified in patients with significant range of motion deficits, as prolonged delay can result in severe, irreversible stiffness [6]. Recommended timing for surgical excision varies, with studies suggesting intervals from six to 12 months and up to one to two years post-injury, depending on the extent of synostosis [7]. Some researchers recommend waiting six to 12 months but not exceeding three years [4]. Preoperative imaging, particularly CT scan with 3D reconstruction, is crucial for evaluating the location and extent of the synostosis and planning the surgical approach [4]. The posterolateral and posterior global approaches are commonly used for proximal radioulnar synostosis, based on Jupiter and Ring's classification [8]. The latter may provide better outcomes, particularly in cases of bony ankylosis at the elbow joint.

Several techniques, including grafts and flaps, have been described to prevent the recurrence of synostosis, though none have emerged as the definitive gold standard. In this case, anconeus muscle interposition was used because it is within the same operative field, eliminating the need for positional changes, thus reducing operative time and technical difficulty. Bell and Benger reported favorable outcomes with vascularized anconeus muscle interposition, with no postoperative complications [9].

Early initiation of postoperative rehabilitation, as employed in this case, likely contributed to the patient's rapid recovery. The use of native muscle within the same operative field not only minimized morbidity but also supported faster functional recovery. Early rehabilitation is crucial in maintaining an optimal arc of motion and preventing recurrent stiffness.

## Conclusions

Despite various techniques available for treating proximal radioulnar synostosis, there is no universally accepted gold standard treatment. The decision to proceed with surgical intervention should be based on multiple factors, including the duration and extent of synostosis and the patient's functional limitations. Early postoperative rehabilitation is vital for preserving the desired range of motion and optimizing recovery. Regular follow-up is also recommended, as this helps identify the risk of recurrence and allows for quick intervention if needed.

## References

[1] Vince KG, Miller JE (1987). Cross-union complicating fracture of the forearm. Part II: children. J Bone Joint Surg Am.

[2] Hastings H 2nd, Graham TJ (1994). The classification and treatment of heterotopic ossification about the elbow and forearm. Hand Clin.

[3] Jupiter JB, Ring D (1998). Operative treatment of post-traumatic proximal radioulnar synostosis. J Bone Joint Surg Am.

[4] Viola RW, Hastings H 2nd (2000). Treatment of ectopic ossification about the elbow. Clin Orthop Relat Res.

[5] Robichaux-Edwards LR, Kunes J (2023). Radioulnar synostosis. StatPearls [Internet].

[6] Pfanner S, Bigazzi P, Casini C, De Angelis C, Ceruso M (2016). Surgical treatment of posttraumatic radioulnar synostosis. Case Rep Orthop.

[7] Friedrich JB, Hanel DP, Chilcote H, Katolik LI (2006). The use of tensor fascia lata interposition grafts for the treatment of posttraumatic radioulnar synostosis. J Hand Surg Am.

[8] Dohn P, Khiami F, Rolland E, Goubier JN (2012). Adult post-traumatic radioulnar synostosis. Orthop Traumatol Surg Res.

[9] Bell SN, Benger D (1999). Management of radioulnar synostosis with mobilization, anconeus interposition, and a forearm rotation assist splint. J Shoulder Elbow Surg.

